# Integrating network pharmacology and experimental validation to investigate the effects and mechanism of Renshen Shouwu decoction for ameliorating Alzheimer’s disease

**DOI:** 10.1080/13880209.2024.2415660

**Published:** 2024-10-17

**Authors:** Jing-jing Liu, Jian-bo Yang, Ying Wang, Xiao-ru Hu, Ya-dan Wang, Li-xing Nie, Feng Wei, Jian-dong Yu, Ling-wen Yao, Bei-lei Xu, Shuang-cheng Ma, Hong-yu Jin

**Affiliations:** a Chinese Academy of Medical Sciences & Peking Union Medical College, Beijing, China; b National Institutes for Food and Drug Control, Beijing, China; c School of Pharmacy, Harbin University of Commerce, Harbin, China

**Keywords:** *Red ginseng*, *Polygoni multiflori*, *Radix Praeparata*, SIRT1, inflammation, apoptosis, aging

## Abstract

**Context:**

The mechanism of Renshen Shouwu Decoction (RSSW) in treating Alzheimer’s disease (AD) remains unknown.

**Objective:**

This study investigates the effects and mechanism of RSSW for ameliorating AD.

**Materials and methods:**

Ten SAMR1 mice and 40 SAMP8 mice were divided into five groups: control (SAMR1), model (SAMP8), positive drug (Donepezil, 1.3 mg/kg/d), and RSSW (Low-dose, 117 mg/kg/d; High-dose, 234 mg/kg/d). Starting from 6 months of age, the medications were administered intragastrically for a total of 60 days. Subsequently, memory improvement in rapidly aging mice was assessed using the novel object recognition test and Morris water maze test. Through the identification of absorbed blood components and analysis of network pharmacology, active ingredients and potential targets involved in the treatment of AD were identified. Finally, AD-related biological indicators were detected using western blotting and ELISA.

**Result:**

Our results demonstrated that RSSW effectively ameliorated memory impairments, inhibited tau hyperphosphorylation, and reduced β-amyloid plaque deposition in SAMP8 mice. Thirty absorbed blood components in RSSW were identified, revealing identified 96 major targets that play a key role in alleviating AD. Notably, the obtained main targets were highly enriched in SIRT1-mediated signaling pathways. Subsequent experimental validation confirmed that RSSW activated the SIRT1/NF-κB, SIRT1/AMPK, and SIRT1/p53 signaling cascades. Nine potential active ingredients were predicted through molecular docking.

**Discussion and conclusions:**

Our research findings suggest the mechanism of RSSW treatment for AD, which ameliorates memory impairments by reducing cortical tissue inflammation and apoptosis.

## Introduction

Alzheimer’s disease (AD), the leading cause of dementia, is characterized by cognitive, functional, and behavioral decline that typically manifests as memory loss, particularly in the context of recent events (Zhang et al. 2021). Despite extensive investigations, the pathogenesis and progression of AD is yet to be comprehensively understood, with hallmark pathological features including β-amyloid plaque deposition, hyperphosphorylated tau neurofibrillary tangles, and neuronal and synaptic loss (Weller and Budson 2018). The increase in the number of individuals with dementia is expected to triple by 2050 (about 150 million), in large part due to an aging population, thereby posing an elevated risk of disability, illness burden, and healthcare expenses (Zhang et al. 2021). In the past two decades, a considerable number of clinical trials, exceeding 100 in total, have been conducted on a global scale to investigate potential treatments for AD. However, the US Food and Drug Administration (FDA) has granted approval to merely four drugs, namely donepezil, galantamine, memantine, and rivastigmine, for their application in clinical AD treatment. It is important to emphasize that these drugs do not confer a definitive therapeutic solution for AD but rather provide partial relief from clinical symptoms (Konecny et al. 2020; Luo et al. 2020). Given the multifactorial nature of AD, pharmacotherapy targeting a single pathological condition may be inadequate, demanding the development of novel and effective anti-AD therapies.

In recent years, there has been an increasing interest in exploring herbal therapy as a potential alternative and complementary intervention for neurodegenerative diseases, specifically AD (Kim et al. 2018; Parvez 2018; Luthra and Roy 2022). *Panax ginseng* C.A. Meyer (Araliaceae) and *Polygonum multiflorum* Thunb (Polygonaceae) are among the herbal medicines that have exhibited potential in this regard (Kim et al. 2018; Gao et al. 2023). Studies have demonstrated that *Panax ginseng* can enhance attention, cognitive processing speed, and working memory, thus augmenting cognitive function and memory in elderly individuals (Lee et al. 2009; Bell et al. 2022; de Oliveira Zanuso et al. 2022). Similarly, *Polygonum multiflorum* has been found to possess neuroprotective properties that can enhance learning and memory ability, reduce brain pathological changes, and alleviate neuroinflammation in mice (Chan et al. 2003; Gao et al. 2023). In China, these two herbs are frequently used in combination as a functional food or alternative and/or complementary medicine to promote overall health and well-being, as well as to protect against age-related diseases (Wan et al. 2015; Parvez 2018). One such formulation is the Renshen Shouwu (RSSW) capsule, a patented traditional Chinese medicine (TCM) consisting of *Red ginseng* and *Polygoni multiflori* Radix Praeparata at a ratio of 2:3, which has been shown to improve memory and is widely used for neurosism and memory deficits (Wan et al. 2015; Li Y et al. 2020). Despite the encouraging outcomes of these studies, the potential roles of RSSW in alleviating AD and its underlying complex mechanisms remain largely unknown.

Network pharmacology is a burgeoning domain of pharmacology that offers a comprehensive and systematic insight into the intricate relationship between drug actions and disease complexities (Li et al. 2023). It shares some basic tenets with traditional Chinese medicine (TCM) theory that emphasizes the importance of integrality and systematics (Yu et al. 2019; Zhao et al. 2023). A growing body of literature indicates that network pharmacology is an effective strategy that can shed light on the multi-dimensional integration synergistic mechanism of TCM (Wu et al. 2022). For instance, the application of ‘compound-target’ network analysis has been instrumental in unravelling the active components and underlying mechanisms of *Gingko biloba* L. (Ginkgoaceae) in treating Alzheimer’s Disease (Singh et al. 2023). Similarly, the ‘compound-protein/gene-disease’ network has been employed to elucidate the action mechanism of Moluodan in chronic atrophic gastritis (Zhou et al. 2022). Nonetheless, this method has its limitations as several previous studies have heavily relied on TCM databases to establish the compound-target map, and the compounds identified may not correspond with the components delivered into the bloodstream, leading to potentially unreliable results (Ding et al. 2019; Ren et al. 2020).

In this work, the therapeutic efficacy of RSSW in AD was evaluated using novel object recognition test, Morris water maze test, and histopathology assay in SAMR1/SAMP8 mice. The absorbed constituents of RSSW were then analyzed by liquid chromatography–tandem mass spectrometry (LC/MS). Through the implementation of the network pharmacology strategy, the molecular targets and pathways involved in the amelioration of AD by RSSW were screened. Finally, the expression levels of the predicted main targets linked with core pathways were confirmed through western blot and ELISA assays. Molecular docking technology was used to predict active ingredients. This study will not only shed light on the anti-AD mechanism of RSSW but also propose a practical methodology for revealing the chemical and pharmacological basis of medicinal plants and prescriptions.

## Materials and methods

### Preparation of RSSW decoction

*Red ginseng* and *Polygoni multiflori* Radix Praeparata were procured in March 2022 from Beijing Tongrentang Pharmacy and verified by Dr. Kang-shuai (National Institutes for Food and Drug Control, Beijing, China) to ensure their authenticity. *Red ginseng* and *Polygoni multiflori* Radix Praeparata were crushed and then passed through a second sieve. The medicinal powder was weighed in a ratio of 2:3. Weighed 300 g of mixed medicinal powder, added 3 L of 70% ethanol (analytical grade, National Pharmaceutical Group Chemical Reagent Co., Ltd.), soaked overnight at room temperature, and then percolated for 24 h. Combined the permeate filtrates and concentrated it to a paste with a relative density of 1.25–1.30. Dried it under reduced pressure at 60 °C to form a dry extract and ground into a fine powder to obtain RSSW. Before LC/MS analysis, the RSSW decoction was diluted to a concentration of 10 mg/mL (w/v, crude drug/water) and subjected to filtration through a 0.22 μm membrane filter.

### Animals and ethical statement

The senescence-accelerated mouse prone 8 (SAMP8), which is characterized by rapid brain aging, displays both natural aging characteristics and AD-like pathological features. The model effectively simulates aging phenotypes, such as learning and memory impairments that occur with age. It is a widely used and well-characterized model for studying AD. Six-month-old male SAMP8 and senescence-accelerated mouse resistant 1 (SAMR1) mouse were procured from Peking University Health Science Center (Certificate number SCXK (Jing) 2021-0013, Beijing, China). All experimental protocols were approved by the Animal Management and Use Committee of Kangtai Medical Laboratory Services Hebei Co., Ltd. (license number: MDL2022-11-15-01). The animal experiments were conducted by Jingjing Liu from November 2022 to February 2023.

The mice were provided with free access to water and a standard diet and were housed in a controlled rearing room maintained at a temperature of 23 °C, a humidity of 60%, and a 12 h light/dark cycle. Following a 3-day adaptation period in the new environment, SAMR1 mice were given oral normal saline (gavage, 0.1 mL/10 g) and were assigned as the control group (*n* = 10). SAMP8 mice were randomly divided into four groups (*n* = 10): the model group received oral normal saline (gavage, 0.1 mL/10 g), the positive drug group received a dose of 1.3 mg/kg of donepezil (gavage), and the RSSW-treated group received oral treatment with 117 mg/kg (low dose group, gavage) and 234 mg/kg (high dose group, gavage) of RSSW. The dose was calculated using the equivalent dose conversion factor based on the surface area. After 60 days of treatment, a behavioral experiment was conducted. Anesthesia was induced with 3% isoflurane and maintained with 1% isoflurane. We strictly followed humane endpoint criteria throughout the animal experiments. Mice that failed to model the disease were humanely euthanized by CO_2_ inhalation following isoflurane anaesthesia. The death was validated by confirming cardiac and respiratory arrest. The mouse euthanasia was executed by Jingjing Liu.

### Novel object recognition test

The memory ability of mice was evaluated using the novel object recognition (NOR) test, which involved measuring their exploration duration of familiar and unfamiliar objects. This test was conducted following the methodology outlined by Garcia-Just et al. (2020). Briefly, individual mice were placed in a 90° two-arm (25 cm long, 20 cm high, 5 cm wide) black chamber for a duration of 5 min, during which no objects were present. On the subsequent day, two identical objects were positioned equidistantly from the perimeter in the central part of the chamber. Interactions with both objects were recorded for 5 min using an overhead digital video camera. On the third day, the mice were once again placed in the chamber, this time with one familiar object from the previous test and one novel object, to assess memory retention. Interactions with both objects were recorded for 5 min, while the spatial location of the objects remained unchanged throughout the testing. After each test, the chamber was cleaned with 70% ethanol to eliminate olfactory cues. The discrimination index was calculated using the formula: Discrimination index = (Exploration time_novel_ − Exploration Time_old_)/(Exploration time_novel_ + Exploration time_old_).

### Morris water maze test

The Morris water maze (MWM) test was also conducted to evaluate the learning ability and memory of mice. In brief, a circular water pool was divided into four equal sections, one of which had a platform. Swimming training, with a maximum of 120 s to find the platform, was conducted four times daily for 5 days. During training, the mice were placed at a random location and then released to locate the hidden platform. After the final trail, 24 h later, the escape latency (time taken to reach the platform) and the frequency of crossing the platform were recorded using a tracking system.

### Preparation of brain tissue samples

After a 24 h duration of conducting the NOR test, the mice died following cardiac blood collection post-anesthesia. Subsequently, the mice underwent transcardial perfusion with ice-cold saline (0.9%) until the fluid exiting the right atrium was completely transparent, eliminating any peripheral blood from the central nervous system vasculature. The cortex tissues were promptly extracted from the brains and stored at a temperature of −80 °C until they were required for further use.

### Preparation of RSSW-containing blood samples

A total of 18 SAMP8 mice were enrolled in the study and fasted overnight with *ad libitum* access to water before the experiment. Mice were randomly assigned to two groups (*n* = 9), namely Group A (RSSW treated group) and Group B (control group). In the RSSW treated group, mice received oral administration (gavage) of RSSW at a dose of 22.73 g/kg. On the other hand, mice in the control group received oral administration of normal saline (0.1 mL/10 g) in the same volume. Blood samples were collected from the abdominal aorta, under anesthesia, using heparinized tubes at three time points: 0.5, 1, and 2 h (6 mice were sacrificed by performing a bilateral thoracotomy at each time point). At the conclusion of the study, all mice were euthanized by performing a bilateral thoracotomy. The animal ethics committee of Kangtai Medical Laboratory Services Hebei Co., Ltd. approved the animal treatment (license number: MDL2022-11-15-01).

The obtained blood samples were centrifuged at 4 °C for 10 min at a speed of 4000 rpm. The supernatant (200 μL) was mixed with 600 μL of methanol (mass spectrometry grade, Merck KGaA), vortexed for 2 min at 4 °C, and subsequently centrifuged at a speed of 10,000 rpm for 10 min. The supernatant was then aspirated and dried under a stream of nitrogen gas. The dried residue was reconstituted in 200 μL of a methanol-water mixture (1:1, v/v) and centrifuged at a speed of 10,000 rpm for 10 min. The resulting supernatant was collected for LC/MS analysis.

### LC/MS analysis

Chromatographic analysis was carried out using a Waters ACQUITY UPLC system (Waters Corporation, Milford, MA, USA). The separation was performed on a Waters ACQUITY UPLC BEH C18 column (2.1 × 100 mm, 1.7 µm) through gradient elution. The mobile phase comprised water with 0.1% formic acid (A) and acetonitrile with 0.1% formic acid (B). The column temperature was set to 35 °C, while the sample chamber temperature was maintained at 4 °C. The gradient elution program was as follows: 0–1 min, 95% A; 1–20 min, 95% A to 5% A; 20–21 min, 5% A to 95% A; 21–22 min, 95% A. The flow rate was set to 0.5 mL/min, and the injection volume was 1 µL.

The SYNAPT™ XS ion mobility time-of-flight (TOF) mass spectrometer, manufactured by Waters Corporation in Milford, MA, USA, was connected to the UPLC. Full scan data were collected under negative ion mode in the range of 50–1500 Da. The capillary voltage was set to 2.5 kV, and the cone voltage was set to 30 V. The desolvation gas flow was maintained at a rate of 800 L/h, and the temperature was set to 500 °C. The cone gas flow rate was set to 50 L/h, and calibration fluids (leucine enkephalin) were used to ensure accuracy and repeatability. MassLynx V4.1 software, developed by Waters Corp. in Milford, MA, USA, was employed for accurate mass determination, composition analysis of precursor ions, and calculation of fragment ions.

### Network pharmacology analysis

#### Acquisition of protein targets of absorbed components

The absorbed components of RSSW were subjected to drug similarity searches using MedChem Studio (version 3.0; Simulations Plus, Inc, Lancaster, CA, USA, 2012), which is known for its efficient prediction of target associations. Only drugs with structural similarity scores exceeding 0.60 were selected to ensure the accuracy of the predicted targets (Luo et al. 2021).

#### Screening of AD-related targets

By utilizing the search query ‘Alzheimer’s disease’, we conducted a search in the DrugBank database available at https://www.drugbank.ca and the OMIM database accessible at http://www.omim.org to identify candidate therapeutic targets associated with AD. The target data from both disease databases were combined, and duplicate values were removed to obtain AD-related targets.

#### Protein–protein interaction data

The targets related to RSSW and AD were uploaded to the STRING (Search Tool for the Retrieval of Interacting Genes/Proteins) database (http://string-db.org/, version 11.0) to predict potential protein-protein interaction (PPI) data. PPI data with confidence scores >0.4 were retained (Luo et al. 2023).

#### Network construction and analysis

A ‘component-target-disease’ network was built by utilizing the PPI data obtained from the STRING database. This network was visualized using Cytoscape software 3.7.1 (https://cytoscape.org/), a popular freely available software for biological network visualization and data integration (Shannon et al. 2003). To assess the topological significance of each node, three key parameters, namely degree centrality (DC), betweenness centrality (BC), and closeness centrality (CC), were employed. The median values of these three topological properties were chosen as the cutoff points.

#### Pathway enrichment performance

By utilizing the DAVID (Database for Annotation, Visualization and Integrated Discovery) system (http://david.abcc.ncifcrf.gov/home.jsp/, version 6.7), the pathways implicated in the action of RSSW on AD were elucidated through the analysis of Kyoto Encyclopedia of Genes and Genomes (KEGG) pathway (KEGG, http://www.genome.jp/kegg/) and gene ontology (GO) enrichment. A significance level of *p* < 0.05 was deemed indicative of high confidence.

#### Molecular docking analysis

A molecular docking study was conducted to explore the interaction between SIRT1 and components of RSSW. The SDF format files containing the components were retrieved from the PubChem database (https://www.ncbi.nlm.nih.gov/pccompound). Additionally, the three-dimensional structure of SIRT1, with a resolution of <2.50 Å, was obtained from the Protein Data Bank (PDB) database (https://www.rcsb.org/). Subsequently, the compound and target files were inputted separately into the CB-Dock2 platform (https://cadd.labshare.cn/cb-dock2/php/index.php) for molecular docking analysis. It is noteworthy that a lower binding energy below −8 indicates a more stable docking conformation and a stronger intrinsic binding capability.

#### Western blot assay

Proteins from brain tissues were extracted using RIPA lysis buffer (MDL, China) that contained a cocktail of protease and phosphatase inhibitors. The protein concentration was then determined using BCA protein assay kits (MDL, China). Proteins (20 µg/lane) were separated on 10% SDS-PAGE and transferred onto a polyvinylidene fluoride (PVDF) membrane. Following the transfer, the membranes were sealed in 5% skim milk for 1 h to block nonspecific binding. The PVDF membrane was then incubated overnight at 4 °C with primary antibodies: anti-tau (phospho T231) (#ab151559 1:5000, Abcam, UK), anti-tau (#ab151559 1:5000, Abcam, UK), anti-SIRT1 (#9475 T 1:1000, CST USA), anti-acetyl-p53(K379) (#2570S 1:1000, CST USA), anti-NF-kB p65 (#8242 1:1000, CST USA), anti-p53 (#32532S 1:1000, CST USA), anti-Bcl-2 (#3498s 1:1000, CST USA), anti-Bax (#2772s 1:1000, CST USA), anti-AMPK (#AF6423 1:1000, AFFINITY USA), anti-p-AMPK (#AF3423 1:1000, AFFINITY USA), anti-acetyl-NF-kB p65(#AF1017 1:1000, AFFINITY USA). After washing with TBST, the membranes were incubated with secondary horseradish peroxidase-conjugated antibodies (1:5000) at room temperature for 1 h. Finally, the protein bands were visualized using an enhanced chemiluminescence (ECL) kit (Bio-Rad, CA, USA) in a gel imager (ChemiScope6100, CLINX Qinxiang, Guangzhou, China) and quantified using Image J software.

#### Enzyme-linked immunosorbent assay (ELISA)

The concentrations of Aβ_1–42_ (JL11386, Jianglai, Shanghai, China), IL-1β, IL-6, and TNF-α (abs552801, abs552805, abs552805, Absin, Shanghai, China) in the cortex were quantified utilizing ELISA kits as per the guidelines provided by the manufacturer. The ELISA kit was equilibrated at room temperature for 20 min. Following this, the standard working solution and cortex tissue lysates were added and immediately mixed with the biotinylated antibody detection working solution. The plate was then incubated at 37 °C for 45 min. After removing the solution, the plate was washed three times. Subsequently, the horseradish peroxidase (HRP) conjugated working solution was added and the plate was incubated at 37 °C for 30 min. After the addition of substrate reagent, the plate was incubated at 37 °C for 15 min. The stop solution was added and the optical density at 450 nm was determined using a microplate reader (Bio-Tek Elx800; BioTek Instruments, Inc., Winooski, VT, USA).

### Statistical analysis

All experiments were conducted with a minimum of three biological replicates and numerical data were reported as mean ± standard deviation (*SD*). Statistical analysis was conducted using two-tailed Student’s *t*-tests or one-way ANOVA with Tukey’s multiple comparison tests with GraphPad Prism software version 9.0. A *p*-value of <0.05 was considered statistically significant.

## Results

### RSSW ameliorated memory retention loss in aged mice

**Figure 1.**
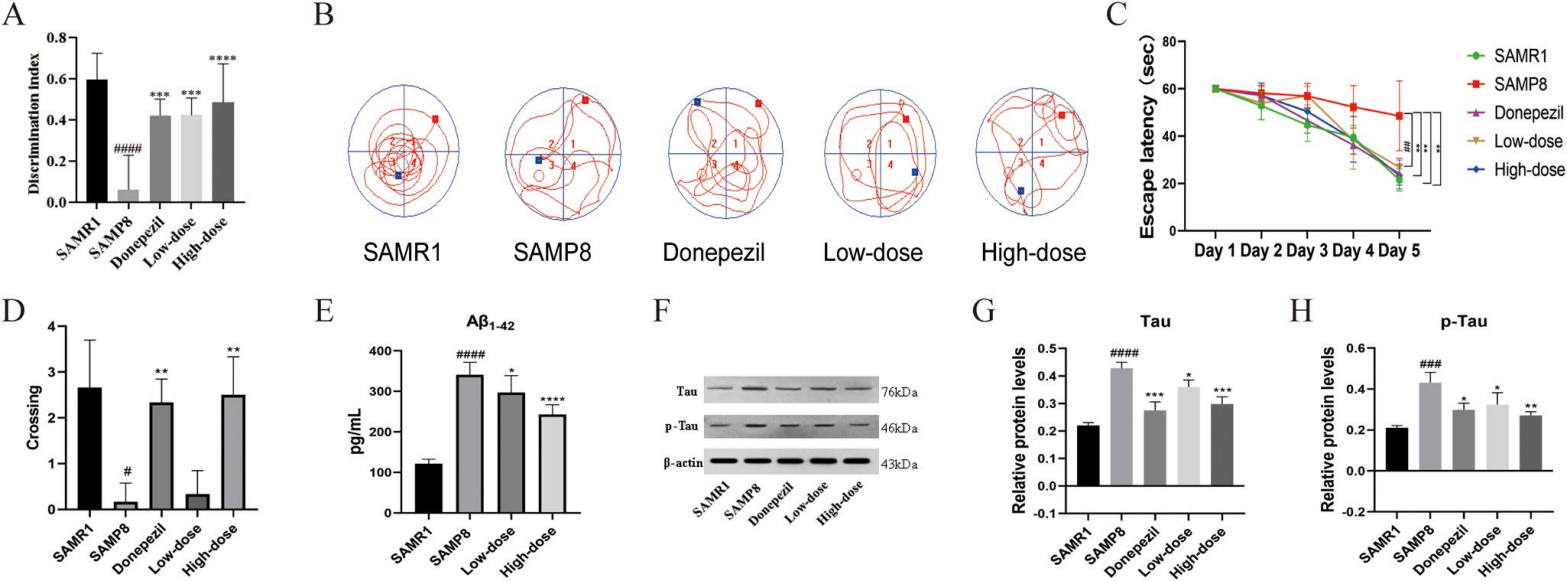
RSSW ameliorates cognitive dysfunction in SAMP8. (A) Discrimination index in novel object recognition, *n* = 6. (B) Movement trajectory in the water maze, *n* = 6. (C) Escape latency in the water maze, *n* = 6. (D) Target crossings in the water maze, *n* = 6. (E) The levels of Aβ_1-42_ in brain cortex tissues quantified by ELISA, *n* = 6. (F) The protein expressions of tau and *p*-tau in brain cortex tissues determined by Western blot, *n* = 3. (G) and (H) The histogram shows the protein expression levels of tau and *p*-tau normalized with β-actin in all groups, *n* = 3. The results were presented as mean ± *SD*, ^#^*p* < 0.05, ^##^*p* < 0.01, ^###^*p* < 0.005, ^####^*p* < 0.001 *vs.* SAMR1 group. *p < 0.05, **p < 0.01, ***p < 0.005, ^****^p < 0.001 *vs.* SAMP8 group.

The NOR and MWM tests were used to evaluate the working memory of mice. Compared to SAMR1 mice, the SAMP8 mice displayed a significant decrease in their discrimination index (*p* < 0.001), indicating a significant impairment in their memory abilities (Figure 1(A)). Following treatment with RSSW, the discrimination indexes significantly improved in all dosing groups. In the Morris water maze test, the mice in the model group exhibited learning and memory deficits compared with the mice in the SAMR1 group, while RSSW at high-dose alleviated the cognitive impairment, as evidenced by the decreased escape latency to find the platform (Figures 1(B,C)) and increased crossing numbers on the platform during the testing after removing the platform (Figure 1(D)). These findings suggest the potential of RSSW to mitigate memory deficits in aged SAMP8 mice.

### RSSW decreased Aβ deposition in aged mice

Amyloid-β (Aβ) constitutes the principal element of senile plaques observed in the brains affected by Alzheimer’s disease (AD) (Shahnur et al. 2021). In this work, we employed an ELISA assay to detect the expression of Aβ_1-42._ As illustrated in Figure 1(E), the cortex of SAMP8 mice exhibited a higher Aβ deposition compared to SAMR1 mice. Following successive RSSW treatment for a duration of 60 days, the levels of Aβ plaques in the cortex of SAMP8 mice noticeably diminished. These observations provide evidence that RSSW possesses an Aβ-lowering effect in SAMP8 mice.

### RSSW reduced tau hyperphosphorylation in aged mice

Tau protein hyperphosphorylation is an initial pathological post-translational modification identified in AD (Basheer et al. 2023). In this study, we used western blot assay to evaluate the expression of tau and *p*-tau 231. As shown in Figures 1(F,G), the expression of tau protein was significantly elevated in SAMP8 mice compared to SAMR1 mice (*p* < 0.001). Following treatment with RSSW, the expression of tau protein significantly decreased. As depicted in Figures 1(F,H), there was a noteworthy increase in the level of *p*-tau 231 in SAMP8 mice relative to SAMR1 mice (*p* < 0.005). Following treatment with RSSW, the expression of *p*-tau 231 significantly decreased. These findings indicated that RSSW attenuated memory impairment by inhibiting tau protein hyperphosphorylation.

### Identification of the absorbed components of RSSW

The UPLC/Q-TOF MS technique was used to detect and identify components in blank plasma samples, RSSW-treated plasma samples, and RSSW samples. Figure S1 and Table 1 showed that a total of 133 components were identified in RSSW decoction, such as ginsenosides, stilbenes, anthraquinones, and phenolic compounds. Furthermore, by comparison of their molecular formulas, fragment ions, and retention times with those of the RSSW sample, 30 absorbed prototypes (Table 2) were identified from the RSSW-treated plasma sample.

**Table 1.** Analysis of the chemical constituents of Renshen Shouwu decoction by LC/MS.

RT (min)	Compound name	Observed m/z	Formula	Mass error (ppm)	Adducts	Fragments	Source
0.57	PM22-25	933.2441	C_46_H_46_O_21_	−1.9	[M-H]^−^	809.23, 703.17, 458.12, 283.06	HSW
0.61	Fallacinol	345.0603	C_16_H_12_O_6_	−3.7	[M + HCOO]^−^	253.05, 179.04	HSW
1.22	Isomer-rumejaposide D	449.1090	C_21_H_22_O_11_	0.19	[M-H]^−^	379.08, 286.05	HSW
4.30	PM1-4	833.2370	C_42_H_42_O_18_	8.5	[M-H]^−^	671.18, 431.10, 254.06	HSW
4.32	Rumejaposide D	495.1155	C_21_H_22_O_11_	2.1	[M + HCOO]^−^	407.08, 259.06, 255.07	HSW
5.71	Physcion-1-*O*-*β*-D-glucopyranoside	445.1146	C_22_H_22_O_10_	1.4	[M-H]^−^	387.05, 283.06, 145.03	HSW
4.21	*β*-D-Glucoside,4-[2,3-dihydro-3-(hydroxymethyl)-5-(3-hydroxypropyl)-7-methoxy-2-yl]-2-methoxypheny	521.2049	C_26_H_34_O_11_	4	[M-H]^−^	359.15, 313.10, 243.06	HSW
0.56	Polygonumnolide E	685.1922	C_37_H_34_O_13_	−0.73	[M-H]^−^	671.18, 416.11, 254.06	HSW
0.74	Polygonumnolides B_1_-B_3_	847.2535	C_43_H_44_O_18_	9.5	[M-H]^−^	707.17, 416.11, 283.06	HSW
4.30	Emodin-8-*O*-(6′-*O*-acetyl)-*β*-D-glucopyranoside	473.1070	C_23_H_22_O_11_	−4.1	[M-H]^−^	269.05, 225.06	HSW
6.32	Emodin 8-*O*-*β*-D-glucoside	431.0982	C_21_H_20_O_10_	−0.3	[M-H]^−^	269.05, 225.06	HSW
6.42	Carboxylemodin	313.0368	C_16_H_10_O_7_	4.4	[M-H]^−^	269.04, 243.06	HSW
7.06	1,6-Dimethylether-emodin	297.0770	C_17_H_14_O_5_	0.4	[M-H]^−^	283.06, 240.04	HSW
7.07	Physcione	283.0624	C_16_H_12_O_5_	4.2	[M-H]^−^	255.02, 240.04	HSW
7.33	Polygonumnolides C_1_-C_4_	671.1779	C_36_H_32_O_13_	1.3	[M-H]^−^	553.10, 458.12, 254.06	HSW
8.56	Chrysophanol	299.0574	C_15_H_10_O_4_	4.2	[M + HCOO]^−^	225.05	HSW
8.57	Emodin-9-anthrone	255.0676	C_15_H_12_O_4_	5	[M-H]^−^	137.02, 109.03	HSW
9.67	2-Acetyl-emodin	311.0573	C_17_H_12_O_6_	3.9	[M-H]^−^	283.06, 269.05	HSW
10.36	Emodin	269.0456	C_15_H_10_O_5_	0.1	[M-H]^−^	241.05, 225.06	HSW
16.95	Isomer-torachrysone-8-*O*-(6′-*O*-acetyl)-*β*-D-glucopyranoside	495.1516	C_22_H_26_O_10_	1.7	[M + HCOO]^−^	359.09, 255.07, 159.04	HSW
0.71	(Isomer) 2,3,5,4′-tetrahydroxystilbene-2,3-*O*-*β*-D-glucopyranoside	567.1673	C_26_H_32_O_14_	−8.2	[M-H]^−^	405.12, 243.07, 225.06	HSW
1.09	7-Acetyl-3,8-dihydroxy-6-methyl-1-naphthyl-*β*-D-glucopyranoside	439.1216	C_19_H_22_O_9_	−6.7	[M + HCOO]^−^	295.06, 273.08, 231.07	HSW
3.00	Multiflorumiside A-I	811.2449	C_40_H_44_O_18_	−0.8	[M-H]^−^	649.19, 487.14, 243.07	HSW
3.07	(Isomer) polygonumoside C/D	827.2405	C_40_H_44_O_19_	0.1	[M-H]^−^	665.19, 467.11, 225.05	HSW
3.33	Multiflorumiside L/K	1217.3835	C_60_H_66_O_27_	9.6	[M-H]^−^	811.25, 646.17, 405.12	HSW
3.39	(Isomer) polygonumoside C/D	827.2412	C_40_H_44_O_19_	1	[M-H]^−^	665.19, 467.11, 225.05	HSW
3.74	7-Hydroxy-3,4-dimethylcoumarin-5-*O*-*β*-D-glucopyranoside	367.1054	C_17_H_20_O_9_	5.4	[M-H]^−^	243.07, 225.06	HSW
3.98	(Isomer) multiflflorumiside A1/B1	809.2331	C_40_H_42_O_18_	4	[M-H]^−^	647.18, 485.12, 267.07	HSW
4.20	Polydatin	389.1274	C_20_H_22_O_8_	8.2	[M-H]^−^	227.07	HSW
4.31	Tetrahydroxystilbene glucoside	405.1196	C_20_H_22_O_9_	1.1	[M-H]^−^	243.07, 225.06	HSW
4.40	2,3,5,4′-Tetrahydroxystilbene-2-*O*-*β*-D-(3-*O*-monogalloylesters)-glucopyranoside	557.1325	C_27_H_26_O_13_	4.4	[M-H]^−^	405.12, 313.06, 243.07	HSW
4.79	Polygonumoside A/B	555.1156	C_27_H_24_O_13_	2.2	[M-H]^−^	393.06, 274.01, 230.06	HSW
5.02	*β*-Glucopyranoside,3-hydroxy-5-[(1*E*)-2-(4-hydroxyphenyl)ethenyl]phenyl,2-(3,4,5-trihydroxybenzoate)	541.1391	C_27_H_26_O_12_	7.3	[M-H]^−^	485.12, 313.06, 169.01	HSW
5.23	Tetrahydroxystilbene-*O*-(caffeoyl)-glucopyranoside	567.1513	C_29_H_28_O_12_	0.9	[M-H]^−^	405.12, 243.06	HSW
5.34	Polygonimitin E	539.1765	C_25_H_32_O_13_	−0.91	[M-H]^−^	489.12, 405.12, 256.04	HSW
5.64	Desoxyrhaponticin	403.1418	C_21_H_24_O_8_	4.8	[M-H]^−^	349.07, 269.05	HSW
5.77	2,3,5,4′-Tetrahydroxystilbene-2-*O*-*β*-D-(2″-*O*-coumaroyl)-glucoside	551.1570	C_29_H_28_O_11_	2	[M-H]^−^	389.10, 242.00	HSW
5.81	Polygonibene E	581.1687	C_30_H_30_O_12_	3.8	[M-H]^−^	405.12, 243.07	HSW
6.08	PM 26-27	889.2514	C_45_H_46_O_19_	−5.2	[M-H]^−^	847.25, 458.12, 254.06	HSW
3.82	(Isomer) 2,3,5,4′-tetrahydroxystilbene-2-*O*-*β*-D-glucopyranoside	405.1172	C_20_H_22_O_9_	−3.5	[M-H]^−^	243.07, 189.06	HSW
3.64	2,3,5,4′-Tetrahydroxystilbene-2,3-*O*-*β*-D-glucopyranoside	567.1673	C_26_H_32_O_14_	−8.2	[M-H]^−^	405.12, 243.07, 225.06	HSW
0.62	Catechin	289.0703	C_15_H_14_O_6_	−4.9	[M-H]^−^	179.08	HSW
6.19	Epicatechin-3-*O*-gallate	441.0843	C_22_H_18_O_10_	3.5	[M-H]^−^	289.07, 169.01	HSW
0.70	3-*O*-Galloyl-procyanidin B_2_	775.1484	C_37_H_30_O_16_	−4.1	[M + HCOO]^−^	589.15, 499.13, 247.06	HSW
6.60	Isomer-procyanidin B_2_	577.1359	C_30_H_26_O_12_	1.3	[M-H]^−^	439.11, 345.08, 182.02	HSW
6.61	Procyanidin B_2_	577.1359	C_30_H_26_O_12_	1.3	[M-H]^−^	451.10, 407.08, 289.07	HSW
0.64	Tricin	329.0647	C_17_H_14_O_7_	−5.9	[M-H]^−^	313.05, 254.06, 161.02	HSW
4.11	D-Lyoniresinol-3-*O*-*β*-D-glucopyranoside	581.2250	C_28_H_38_O_13_	1.7	[M-H]^−^	549.16, 387.11, 253.01	HSW
4.87	Quercetin-3-*O*-rhamnoside	447.0955	C_21_H_20_O_11_	5	[M-H]^−^	361.07, 285.04	HSW
5.56	Dihydroquercetin	303.0524	C_15_H_12_O_7_	4.6	[M-H]^−^	153.02, 125.02	HSW
6.96	Kaempferol	285.0417	C_15_H_10_O_6_	4.3	[M-H]^−^	193.01, 125.02	HSW
7.83	Quercetin	301.0373	C_15_H_10_O_7_	6.5	[M-H]^−^	285.04, 257.05, 179.02	HSW
18.27	1,3-Dihydroxy-6,7-dimethylxanthone-1-*O*-*β*-D-glucopyranoside	463.1608	C_22_H_26_O_8_	−0.4	[M + HCOO]^−^	259.06, 137.02	HSW
13.57	LPC16:0	540.3309	C_24_H_50_NO_7_P	1.5	[M + HCOO]^−^	480.31, 311.17, 255.23	HSW
7.81	Phosphatidylinositollyso 18:1	597.2999	C_27_H_51_O_12_P	−6.9	[M-H]^−^	417.02	HSW
13.57	Phosphatidylethanolaminelyso 18:0	480.3096	C_23_H_48_NO_7_P	1.2	[M-H]^−^	213.81	HSW
1.13	2,3,6-Trimethylnaphthalene	169.1018	C_13_H_14_	3.7	[M-H]^−^	155.09	HSW
5.37	Compound Ra_1_	931.5208	C_46_H_78_O_16_	−6.9	[M + HCOO]^−^	619.42, 603.43, 457.37	RS
5.38	Ginsenoside Re_4_	977.5321	C_47_H_80_O_18_	−0.6	[M + HCOO]^−^	829.46, 769.47, 653.43, 311.20	RS
5.50	Pseudoginsenoside RT_5_	699.4335	C_36_H_62_O_10_	1.4	[M + HCOO]^−^	491.37	RS
5.60	Ginsenoside Re	991.5472	C_48_H_82_O_18_	−1.1	[M + HCOO]^−^	783.49, 637.43, 475.38, 391.29, 179.06	RS
11.00	Protopanaxatriol	521.3861	C_30_H_52_O_4_	2.5	[M + HCOO]^−^	475.37, 391.27	RS
5.62	Ginsenoside Rf	845.4889	C_42_H_72_O_14_	−1.8	[M + HCOO]^−^	737.43, 635.42, 475.38, 391.29	RS
7.04	Ginsenoside Rg_1_	845.4902	C_42_H_72_O_14_	0.4	[M + HCOO]^−^	799.49, 475.38, 391.29	RS
6.62	Pseudoginsenoside Rs_1_	1033.5598	C_50_H_84_O_19_	0.9	[M + HCOO]^−^	825.50, 781.47, 763.46	RS
6.73	Ginsenoside F_1_	683.4389	C_36_H_62_O_9_	1.9	[M + HCOO]^−^	587.40, 475.38, 391.29, 149.05	RS
6.82	Notoginsenoside S	1387.6843	C_63_H_106_O_30_	6.6	[M + HCOO]^−^	1059.57, 867.51, 785.47, 459.38	RS
6.84	Ginsenoside Rh_7_	681.4218	C_36_H_60_O_9_	−0.2	[M + HCOO]^−^	473.36, 619.42	RS
6.88	Ginsenoside Re_6_	913.5094	C_46_H_76_O_15_	−7.9	[M + HCOO]^−^	781.47, 769.47, 587.32	RS
7.04	Compound Mc	799.4844	C_41_H_70_O_12_	−0.7	[M + HCOO]^−^	621.44, 459.38, 409.31	RS
7.20	Notoginsenoside Fa	1285.6404	C_59_H_100_O_27_	−2.3	[M + HCOO]^−^	1059.57, 943.53, 781.47, 459.38	RS
7.21	Gypenoside XDII	945.5475	C_48_H_82_O_18_	4.9	[M-H]^−^	341.11, 161.05	RS
7.22	Ginsenoside Rb_1_	1153.5986	C_54_H_92_O_23_	−2.2	[M + HCOO]^−^	945.54, 783.49, 621.44, 459.38	RS
7.26	Notoginsenoside R_2_	815.4792	C_41_H_70_O_13_	−0.7	[M + HCOO]^−^	671.40, 603.42, 475.38, 149.05	RS
7.39	Ginsenoside Ra_1_	1255.6298	C_58_H_98_O_26_	−2.4	[M + HCOO]^−^	1077.59, 1047.57, 943.53, 459.38, 131.03	RS
7.46	Ginsenoside Ra_3_	1285.6405	C_59_H_100_O_27_	−2.3	[M + HCOO]^−^	1077.59, 945.54, 783.49, 621.44, 149.05	RS
7.51	Mal-ginsenoside Rd	1031.5389	C_51_H_84_O_21_	−4.2	[M-H]^−^	943.53, 783.49, 603.43, 459.38	RS
7.52	Ginsenoside Ro	955.4898	C_48_H_76_O_19_	−1.1	[M-H]^−^	793.44, 631.37, 587.40, 455.36, 411.37	RS
15.90	Oleanolic acid	455.3537	C_30_H_48_O_3_	1.4	[M-H]^−^	411.37	RS
7.55	Ginsenoside F_1_	683.4372	C_36_H_62_O_9_	−0.6	[M + HCOO]^−^	587.40, 475.38, 391.29	RS
7.59	Ginsenoside F_2_	829.4949	C_42_H_72_O_13_	−0.7	[M + HCOO]^−^	765.48, 637.43, 601.41, 537.34, 475.38	RS
7.61	Ginsenoside Rb_2_	1123.5871	C_53_H_90_O_22_	−3.1	[M + HCOO]^−^	945.54, 915.53, 897.52, 783.49, 311.10	RS
7.73	Mal-ginsenoside Rb_1_	1193.5955	C_57_H_94_O_26_	−0.5	[M-H]^−^	1089.59, 945.54, 781.47, 459.38	RS
8.10	Ginsenoside Rd	991.5463	C_48_H_82_O_18_	−2	[M + HCOO]^−^	667.44, 621.44, 459.38	RS
13.35	Compound CK	667.4422	C_36_H_62_O_8_	−0.7	[M + HCOO]^−^	621.35, 459.38	RS
8.87	Pseudo-ginsenoside RC_1_	1033.5567	C_50_H_84_O_19_	−2.1	[M + HCOO]^−^	927.53, 769.47, 571.33	RS
8.93	Compound IX	961.5372	C_47_H_80_O_17_	−0.6	[M + HCOO]^−^	767.49, 489.32, 473.33	RS
9.29	Quinquenoside III	987.5520	C_50_H_84_O_19_	−1.4	[M-H]^−^	897.52, 783.49, 621.44, 559.33	RS
9.98	Zingibroside R1	793.4360	C_42_H_66_O_14_	−2.5	[M-H]^−^	775.43, 613.75, 595.36, 437.34	RS
10.53	Pseudo-ginsenoside Rp1	763.4312	C_41_H_64_O_13_	5	[M-H]^−^	631.39, 569.38, 453.34	RS
10.68	20(*S*)-Ginsenoside Rg3	829.4937	C_42_H_72_O_13_	−2.2	[M + HCOO]^−^	621.44, 537.34, 161.05	RS
11.36	Oleanolic acid-3-*O*-glucuronide	631.3855	C_36_H_56_O_9_	0.6	[M-H]^−^	613.37, 455.35	RS
11.86	Ginsenoside Rs_3_	871.5068	C_44_H_74_O_14_	0.8	[M + HCOO]^−^	621.44, 519.33, 389.34	RS
11.93	Oleanolic acid-28-*O*-*β*-D-glucopyranoside	663.4148	C_36_H_58_O_8_	5.2	[M + HCOO]^−^	441.34, 371.26, 351.23	RS
15.40	Ginsenoside Rh_3_	649.4328	C_36_H_60_O_7_	1.1	[M + HCOO]^−^	441.3733	RS
16.87	Ginsenoside Rh_5_	697.4523	C_38_H_65_O_11_	5	[M + HCOO]^−^	490.4017	RS
19.19	(Isomer)ginsenoside Rg_2_	783.4925	C_42_H_72_O_13_	3.2	[M-H]^−^	747.69, 621.44, 475.38, 163.06	RS
12.84	Ginsenoside Rg_5_	811.4844	C_42_H_70_O_12_	0	[M + HCOO]^−^	765.48	RS
9.54	Ginsenoside Rg_6_	811.4844	C_42_H_70_O_12_	0	[M + HCOO]^−^	765.48, 374.24	RS
9.77	Ginsenoside Rk_1_	811.4844	C_42_H_70_O_12_	0	[M + HCOO]^−^	765.48	RS
10.87	Ginsenoside Rg_2_	829.4900	C_42_H_72_O_13_	−5.9	[M + HCOO]^−^	783.49	RS
7.47	Ginsenoside LXXV	829.4900	C_42_H_72_O_13_	−5.9	[M + HCOO]^−^	783.49	RS
7.40	Ginsenoside Rc	1123.5890	C_53_H_90_O_22_	−3.1	[M + HCOO]^−^	1107.58	RS
9.42	Kahiricoside V	797.4708	C_42_H_70_O_14_	2.6	[M-H]^−^	751.46	RS
9.63	Rubiarboside G	797.4708	C_42_H_70_O_14_	2.6	[M-H]^−^	751.46, 593.13	RS
6.26	Torachrysone-8-*O*-*β*-D-glucopyranoside	407.1387	C_20_H_24_O_9_	9.7	[M-H]^−^	245.08, 215.04	HSW
4.15	Altechromone A	189.0571	C_11_H_10_O_3_	7.2	[M-H]^−^	147.04, 124.02	HSW
14.40	3-Tetradecanone	257.2126	C_14_H_28_O	1.4	[M + HCOO]^−^	197.19	HSW
13.54	Amauromine	507.2737	C_32_H_36_N_4_O_2_	−4.5	[M-H]^−^	480.31, 311.17	RS
0.55	Grossamide	623.2412	C_36_H_36_N_2_O_8_	2.2	[M-H]^−^	269.05, 416.11	HSW
4.92	*trans*-*N*-Caffeoyltyramine	298.1115	C_17_H_17_NO_4_	10	[M-H]^−^	227.07, 169.08	HSW
5.70	*N*-*trans*-Feruloyltyramine	312.1254	C_18_H_19_NO_4_	4.1	[M-H]^−^	274.01, 178.05, 123.05	HSW
0.59	Citric acid	191.0197	C_6_H_8_O_7_	−0.1	[M-H]^−^	128.04	HSW RS
0.60	3-Methylglutaconic acid	143.0360	C_6_H_8_O_4_	7.4	[M-H]^−^	129.02, 78.96	HSW
0.60	Vanillic acid	167.0351	C_8_H_8_O_4_	4.19	[M-H]^−^	137.03	HSW RS
0.67	Butanedioic acid	163.0263	C_4_H_6_O_4_	9.2	[M + HCOO]^−^	71.01	HSW
0.84	Gallicacid-*O*-glucoside	331.0675	C_13_H_16_O_10_	1.4	[M-H]^−^	169.01, 125.02	HSW
1.02	Gallic acid	169.0148	C_7_H_6_O_5_	3.5	[M-H]^−^	125.02	HSW
1.43	Protocatechuicacid-*O*-glucoside	315.0734	C_13_H_16_O_9_	4.1	[M-H]^−^	195.03, 153.02	HSW
2.85	2-Vinyl-1*H*-indole-3-carboxylic acid	186.0576	C_11_H_9_NO_2_	8.5	[M-H]^−^	142.07	HSW
18.82	Tetradecanoic acid ethyl ester	255.2341	C_16_H_32_O_2_	4.6	[M-H]^−^	205.16	HSW
19.68	Octadecanoic acid methyl ester	297.2826	C_19_H_38_O_2_	9.1	[M-H]^−^	241.05, 119.95	HSW
19.87	Hexadecanoic acid ethyl ester	283.2652	C_18_H_36_O_2_	3.4	[M-H]^−^	183.01, 163.11	HSW
0.71	Furoic acid	111.0093	C_5_H_4_O_3_	9.9	[M-H]^−^	87.01	HSW
0.62	Quinic acid	191.0553	C_7_H_12_O_6_	−1.6	[M-H]^−^	173.01	RS
2.21	Dimethylcitric acid	219.0518	C_8_H_12_O_7_	5.9	[M-H]^−^	195.90, 168.89, 145.93, 111.01	HSW
19.37	Mevinic acid	407.2464	C_23_H_36_O_6_	7.4	[M-H]^−^	349.17, 325.18	HSW
0.74	L-Pyroglutamic acid	128.0349	C_5_H_7_NO_3_	0.8	[M-H]^−^	82.03	HSW
0.60	Lactic acid	89.0238	C_3_H_6_O_3_	−1.1	[M-H]^−^	89.02	HSW
16.01	16-Hydroxyhexadecanoic acid	271.2258	C_16_H_32_O_3_	−5.5	[M-H]^−^	128.91	HSW
8.97	Methyl-2-hydroxyhexadecanoate	331.2512	C_17_H_34_O_3_	6.7	[M + HCOO]^−^	255.23, 227.24	HSW
0.59	Verbaspinoside	683.2233	C_30_H_38_O_15_	6.7	[M + HCOO]^−^	515.12, 353.07	HSW
3.21	Mitomycin C	333.1197	C_15_H_18_N_4_O_5_	−0.6	[M-H]^−^	170.06, 142.03	HSW

RS: *Red ginseng*; HSW: *Polygoni multiflori* Radix Praeparata.

**Table 2.** The absorbed constituents in mice blood after oral administration of RSSW decoction.

RT (min)	Compound name	Observed m/z	Formula	Mass error (ppm)	Adducts	Fragments
0.59	Citric acid	191.0197	C_6_H_8_O_7_	−0.1	[M-H]^−^	128.04
0.61	Catechin	289.0724	C_15_H_14_O_6_	2.1	[M-H]^−^	273.08, 181.05
1.11	2,3,6-Trimethylnaphthalene	169.1018	C_13_H_14_	3.7	[M-H]^−^	155.09
1.43	Protocatechuic acid-*O*-glucoside	315.0728	C_13_H_16_O_9_	2.2	[M-H]^−^	195.03, 153.02
2.86	2-Vinyl-1*H*-indole-3-carboxylic acid	186.06	C_11_H_9_NO_2_	−0.8	[M-H]^−^	142.07
4.17	Altechromone A	189.0574	C_11_H_10_O_3_	8.8	[M-H]^−^	147.04, 124.02
4.30	Tetrahydroxystilbene glucoside	405.1196	C_20_H_22_O_9_	0.5	[M-H]^−^	243.07, 189.06
5.37	Pseudoginsenoside RT5	699.4336	C_36_H_62_O_10_	1.5	[M + HCOO]^−^	637.43, 491.37
5.63	Ginsenoside Rf	845.4891	C_42_H_72_O_14_	−1.6	[M + HCOO]^−^	737.43, 635.42, 475.38, 391.29
6.17	Epicatechin-3-*O*-gallate	487.0904	C_22_H_18_O_10_	4.5	[M + HCOO]^−^	289.07, 169.01
6.32	Emodin 8-*O*-*β*-D-glucoside	431.1000	C_21_H_20_O_10_	3.8	[M-H]^−^	269.05, 225.06
6.83	Ginsenoside Rh_7_	681.4218	C_36_H_60_O_9_	−0.2	[M + HCOO]^−^	473.36, 619.42
6.97	Kaempferol	285.0422	C_15_H_10_O_6_	6	[M-H]^−^	193.01, 125.02
7.20	Ginsenoside Rb_1_	1153.5986	C_54_H_92_O_23_	−2.9	[M + HCOO]^−^	945.54, 783.49, 621.44, 459.38
7.50	Ginsenoside Ro	955.492	C_48_H_76_O_19_	1.3	[M-H]^−^	793.44, 613.37, 587.40
7.59	Ginsenoside Rb_2_	1123.5877	C_53_H_90_O_22_	−2.5	[M + HCOO]^−^	945.54, 915.53, 897.52, 783.49, 311.10
7.83	Quercetin	301.0373	C_15_H_10_O_7_	6.5	[M-H]^−^	285.04, 257.05, 179.02
8.09	Ginsenoside Rd	991.5441	C_48_H_82_O_18_	−4.3	[M + HCOO]^−^	911.54, 783.49, 621.44, 375.29, 179.06
8.56	Chrysophanol	299.0573	C_15_H_10_O_4_	4.1	[M + HCOO]^−^	225.05
8.57	Emodin-9-anthrone	255.0675	C_15_H_12_O_4_	4.8	[M-H]^−^	137.02, 109.03
8.95	Methyl 2-hydroxyhexadecanoate	331.2487	C_17_H_34_O_3_	−1	[M + HCOO]^−^	255.23, 227.24
9.98	Zingibroside R1	793.4365	C_42_H_66_O_14_	−1.8	[M-H]^−^	775.43, 613.75, 595.36, 437.34
10.36	Emodin	269.0477	C_15_H_10_O_5_	8.1	[M-H]^−^	241.05, 225.06
10.69	20(*S*)-Ginsenoside Rg_3_	829.4947	C_42_H_72_O_13_	−1	[M + HCOO]^−^	605.44, 443.39
12.00	Oleanolic acid-28-*O*-*β*--D-glucopyranoside	663.4161	C_36_H_58_O_8_	7.1	[M + HCOO]^−^	441.34, 371.26, 351.23
13.16	Ginsenoside Rg_4_	811.4827	C_42_H_70_O_12_	−2.7	[M + HCOO]^−^	603.43, 441.37
13.36	Compound CK	667.4450	C_36_H_62_O_8_	3.6	[M + HCOO]^−^	621.35, 459.38
14.46	3-Tetradecanone	257.2136	C_14_H_28_O	5.4	[M + HCOO]^−^	197.19
15.52	Ginsenoside Rh_3_	649.4357	C_36_H_60_O_7_	5.5	[M + HCOO]^−^	441.3733
16.85	Ginsenoside Rh_5_	697.4523	C_37_H_64_O_9_	5	[M + HCOO]^−^	490.4017

### The mechanisms of action of RSSW on AD via network pharmacology

#### Putative targets of RSSW

All the absorbed prototypes of RSSW were used in the process of target screening. By employing MedChemStudio, a total of 520 putative targets involved in the therapeutic process of RSSW were acquired.

#### AD-related targets

A total of 14 targets related to AD were screened from the DrugBank database, while 122 targets relevant to AD were obtained from the OMIM database. After eliminating duplicates, 132 AD-related targets were identified and reserved.

#### Network and pathway analysis

To uncover the potential connections between RSSW and AD, we constructed a PPI network that encompasses interactions between RSSW-related targets and AD-related targets. This network consists of 606 nodes and 7658 edges. Using the median value of node degree (20.5) in this network as a cutoff point, 303 nodes were identified as hubs. After evaluating the three topological parameters of each hub in the network, we identified 96 nodes as major hubs based on meeting the screening criteria (DC > 33, BC > 0.0023, CC > 0.4150). Among these major hubs, 73 were RSSW-related targets, 19 were AD-related targets, and four were both targets of RSSW and AD. Detailed information about these major hubs is provided in Table S1.

**Figure 2.**
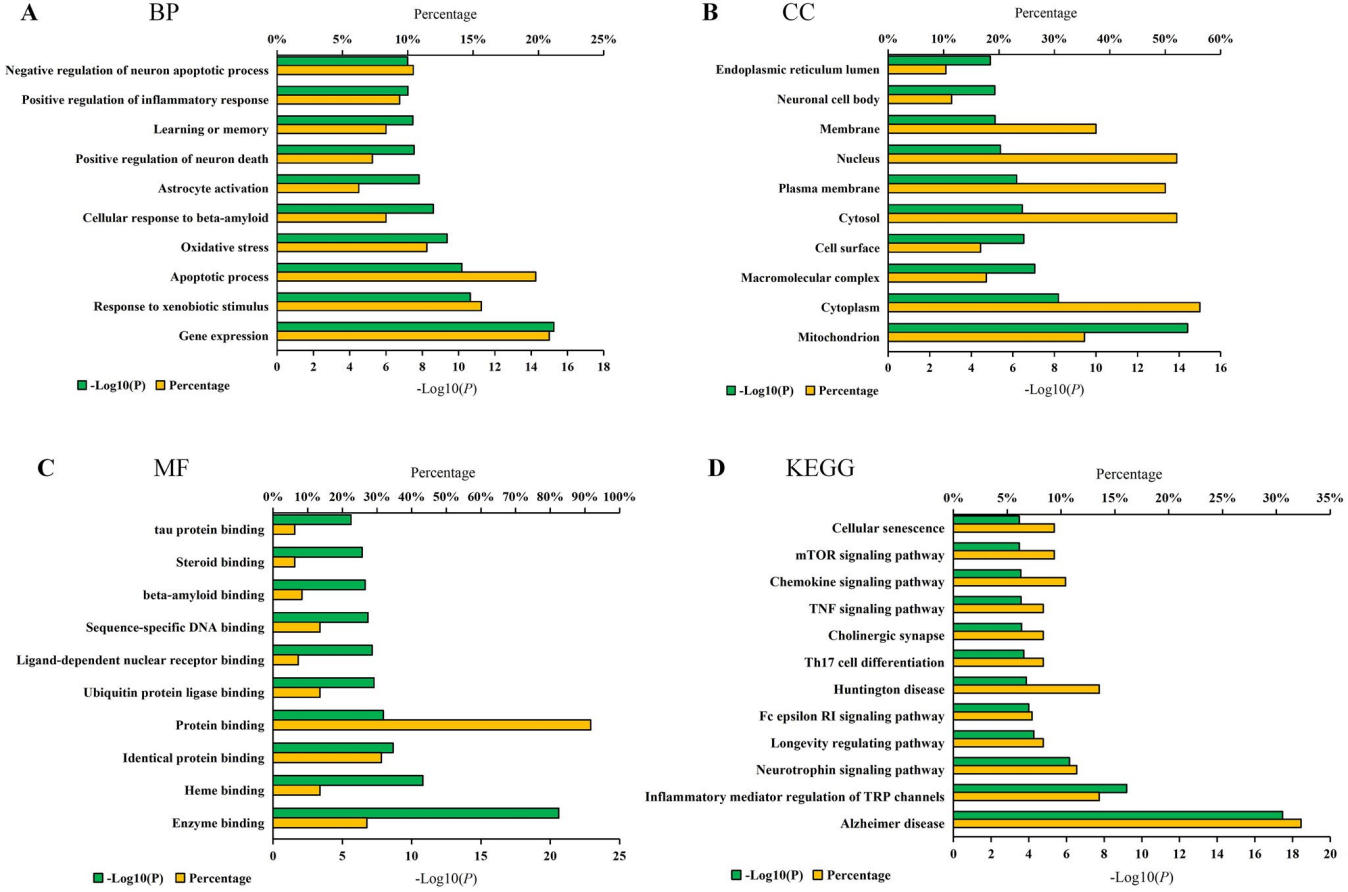
GO term performance and pathway enrichment analysis of the major hubs. (A) GO term performance by biological process (BP); (B) GO term performance by cellular component (CC); (C) GO term performance by molecular function (MF); and (D) pathway enrichment analysis by KEGG. The ordinate stands for GO terms or the main pathways, the primary abscissa stands for minus log10(P), and the secondary abscissa stands for the percentage of major hubs involved in the corresponding GO terms or the main pathways out of total major hubs.

Next, we performed a GO enrichment analysis to better understand the biological functions of these major hubs. Figures 2(A–C) illustrate the top 10 significant GO entries for biological processes (BP), cellular components (CC), and molecular functions (MF). The enriched BP ontologies were dominated by gene expression, response to xenobiotic stimulus, apoptotic process, oxidative stress, cellular response to β-amyloid, astrocyte activation, positive regulation of neuron death, learning or memory, positive regulation of inflammatory response, and negative regulation of neuron apoptotic process (Figure 2(A)). The enriched CC ontologies included mitochondrion, cytoplasm, macromolecular complex, cell surface, cytosol, plasma membrane, nucleus, membrane, neuronal cell body, and endoplasmic reticulum lumen (Figure 2(B)). Additionally, the significantly enriched molecular functions included enzyme binding, heme binding, identical protein binding, protein binding, ubiquitin protein ligase binding, ligand-dependent nuclear receptor binding, sequence-specific DNA binding, β-amyloid binding, steroid binding, and tau protein binding (Figure 2(C)).

**Figure 3.**
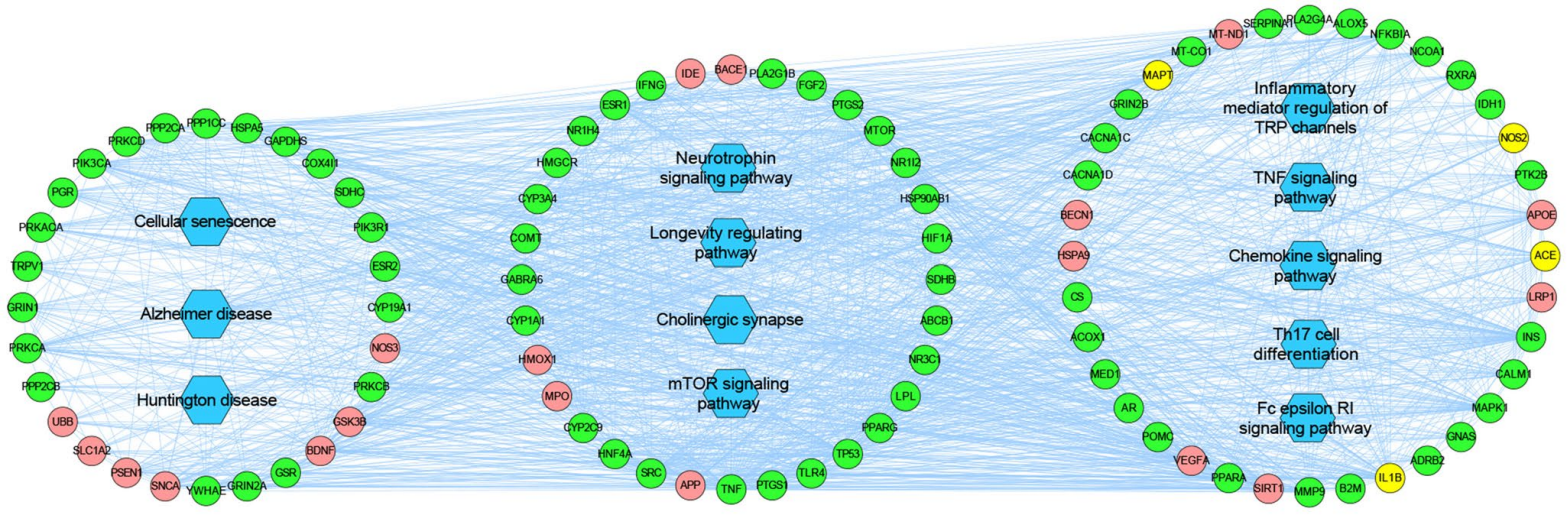
Major hubs-major pathways PPI network. Round green nodes represent putative targets of RSSW; round red nodes represent AD associated targets; round yellow nodes represent both RSSW targets and AD associated targets; blue rectangles represent top 12 pathways from enrichment analysis of major hubs; edges represent interactions among RSSW putative targets, AD associated targets, and main pathways.

To elucidate the specific signaling pathways influenced by the major hubs, a KEGG pathway enrichment analysis was performed. Figure 2(D) displays the top 10 pathways obtained from the analysis. These pathways were categorized into three main functional modules based on functional annotation. The largest module was concentrated on inflammation, including inflammatory mediator regulation of TRP channels, Fc epsilon RI signaling pathway, Th17 cell differentiation, TNF signaling pathway, and Chemokine signaling pathway. The second module was related to the nervous system, specifically neurotrophin signaling pathway, longevity regulating pathway, cholinergic synapse, and mTOR signaling pathway. The smallest module was sorted as senescence and aging, comprising cellular senescence, Alzheimer’s disease, and Huntington disease. We created a network diagram that displayed the interactions among the active constituents of RSSW, main RSSW-related targets, and main pathways (Figure 3). Notably, the KEGG pathway analysis exhibited a remarkable enrichment of Alzheimer’s disease, the longevity regulating pathway, and cellular senescence. Further analysis uncovered a substantial involvement of SIRT1-mediated signaling within these pathways, potentially exerting a pivotal influence on the anti-aging or anti-AD effects of RSSW. Additionally, the molecular docking analysis revealed that nine components (Table S2 and Figure S2), including ginsenoside Rb_2_, epicatechin-3-*O*-gallate, kaempferol, catechin, quercetin, 2,3,6-trimethylnaphthalene, emodin anthrone, ginsenoside Rg4, and zingibroside R1 displayed good affinities towards SIRT1, indicating their potential as key active constituents responsible for the anti-Alzheimer’s disease effects of RSSW.

### RSSW ameliorates AD via activating SIRT1-mediated signaling pathways

**Figure 4.**
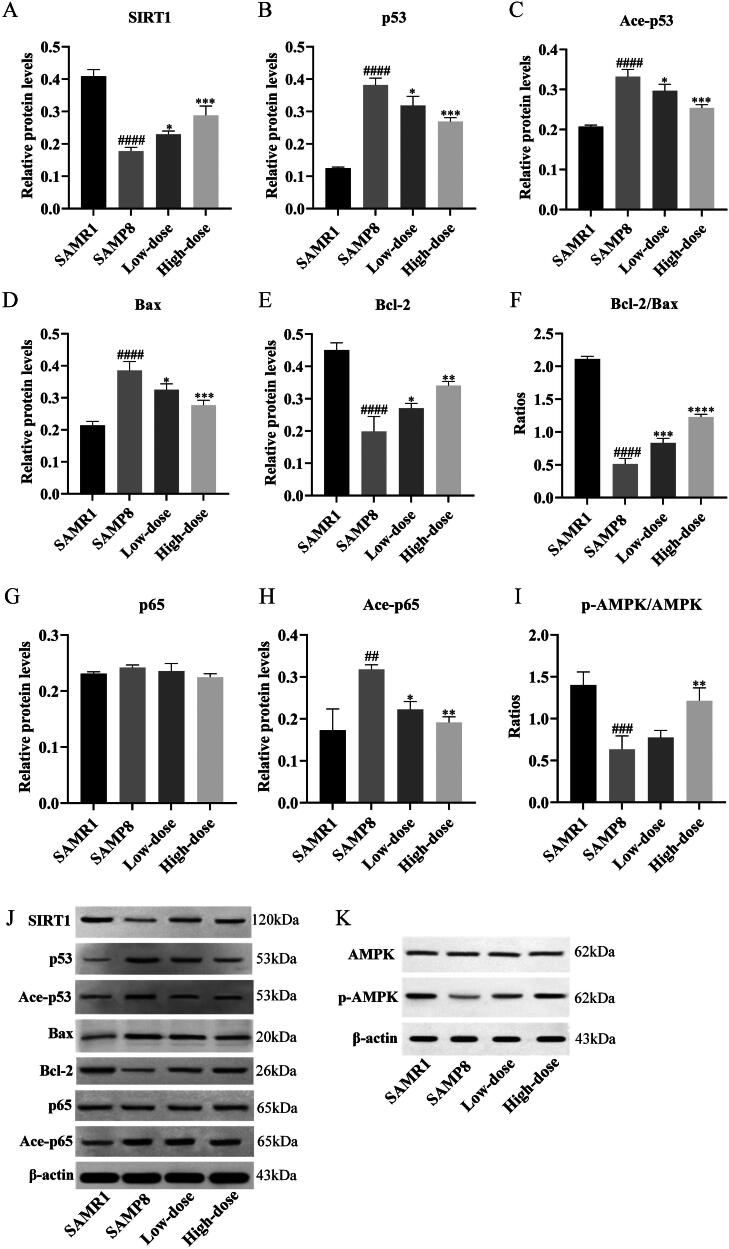
RSSW ameliorates AD *via* activating SIRT1-mediated signaling pathways. (A–I) The histogram shows the protein expression levels of SIRT1, p53, Ace-p53, Bax, Bcl-2, Bcl-2/Bax, p65, Ace-p65, p-AMPK/AMPK (normalized with β-actin), *n = *3. (J,K) The protein expression of SIRT1, p53, Ace-p53, Bax, Bcl-2, p65, Ace-p65, AMPK, and p-AMPK were determined by western blots.

**Figure 5.**
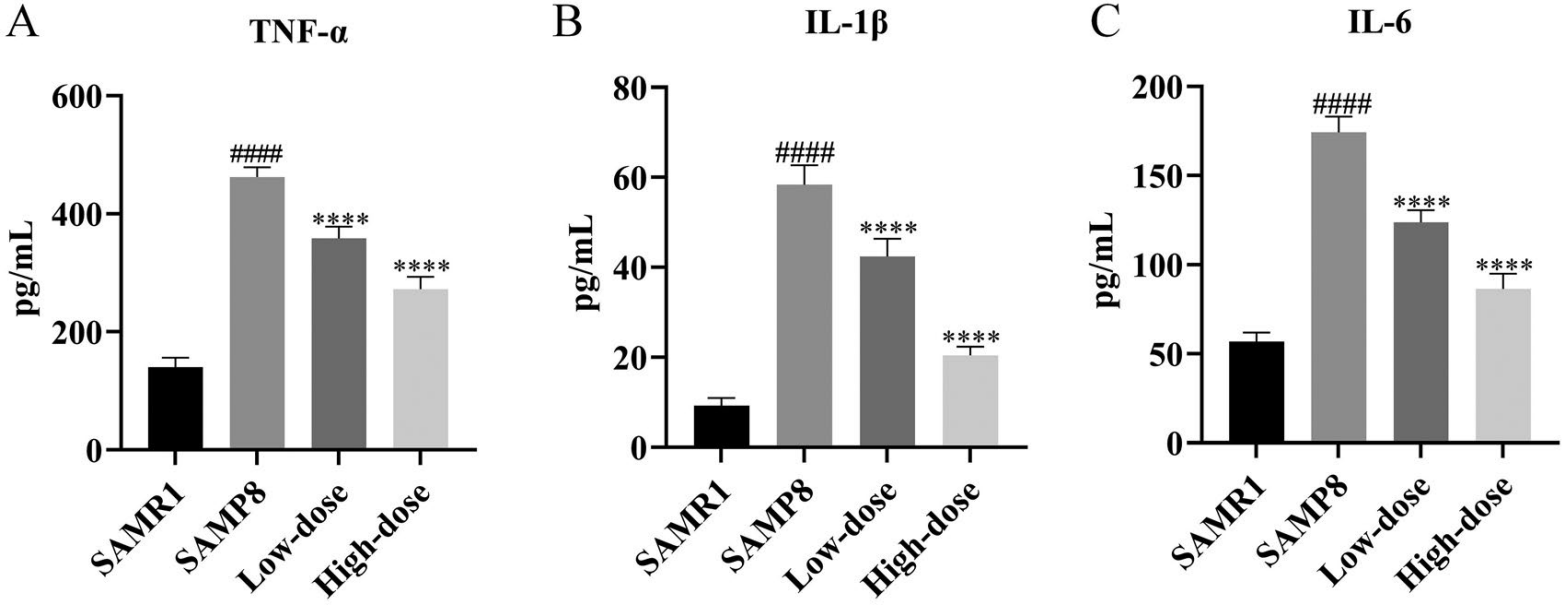
The levels of TNF-α (a), IL-1β (B), and IL-6 (C) in mouse brain tissues quantified by ELISA.

The expression levels of proteins involved in SIRT1-mediated signaling pathways were evaluated using western blot analysis. As depicted in Figure 4, the protein expression of SIRT1 was noticeably lower in SAMR8 mice (model) compared to SAMR1 mice (control). However, following RSSW treatment, the levels of SIRT1 protein significantly increased. Meanwhile, the phosphorylation of AMPK was also visibly enhanced with the increase of RSSW dose, indicating that RSSW treatment could induce cellular autophagy in SAMR8 mice by activating the SIRT1/AMPK signaling pathway (DiNicolantonio et al. 2022). In addition, RSSW down-regulated the levels of p53, Ace-p53, and Bax, while up-regulating Bcl-2 protein expression. This resulted in a decreased ratio of Bax to Bcl-2, suggesting that RSSW treatment could reduce neuronal apoptosis in SAMR8 mice by regulating the SIRT1/p53 signaling pathway (Hemann and Lowe 2006). Furthermore, the protein expressions of acetylated NF-κB p65 (Ace-p65) were significantly decreased in SAMR8 mice after RSSW treatment, while no changes were observed in p65 expression. ELISA results revealed that the levels of multiple cytokines, including IL-1β, IL-6, and TNF-α, were higher in the model group than in the control group (Figure 5). However, their levels were significantly decreased after RSSW treatment. These results indicated that RSSW treatment could alleviate brain inflammation in SAMR8 mice *via* the SIRT1/NF-κB signaling pathway (Yeung et al. 2004).

## Discussion

Sirtuins, a class of NAD^+^ dependent enzymes, play a crucial role in regulating various cellular pathways and have been closely linked to the aging process and age-related diseases. Recently, there has been a growing interest in understanding the importance of sirtuins in the context of AD. A study reveals that the increased expression of SIRT1 directly reduced the presence of amyloid plaques, whereas the depletion of SIRT1 enhanced plaque formation (Donmez 2012). Moreover, it has been observed that SIRT1 can directly deacetylate Tau proteins, making them more susceptible to ubiquitin ligases (Min et al. 2010). As a result, these proteins undergo degradation into multiple residues, thereby impeding the formation of neurofibrillary tangles and impeding the spread of Tau-induced pathology *in vivo* (Min et al. 2018).

Molecular docking results of SIRT1 and components of RSSW revealed that nine components exhibited a stronger intrinsic binding capability. These compounds primarily interact with proteins through hydrogen bonds, van der Waals forces, and π-π interactions. Among them, catechins, kaempferol, ginsenosides Rb_2_, and quercetin had relatively high binding energies to proteins. These compounds could form hydrogen bonds with Q345, N346, K444, and V445, enabling them to establish strong interactions with proteins. However, the binding energy between ginsenoside Rd and the protein was only 5.6 kcal/mol, and only a few residues could interact with the protein.

The research findings (Chen et al. 2005) determined that SIRT1 plays a protective role against microglia-dependent β-amyloid toxicity by hindering the NF-κB signaling pathway, resulting in a reduction of neuroinflammation. The interaction between SIRT1 and the RelA/p65 subunit of NF-κB leads to the deacetylation of lysine residue 310 on the RelA/p65 protein. The acetylation of p65 enhances the transcriptional activity of the NF-κB complex, thus the deacetylation mediated by SIRT1 acts to inhibit NF-κB signaling (Yeung et al. 2004). NF-κB facilitates the transcription and translation of the NLRP3 inflammasome molecules, leading to the release of inflammatory cytokines (Boaru et al. 2012). Consequently, the reduced levels of Ace-p65 and cytokine release suggested that RSSW effectively suppressed neuroinflammation *via* modulating the SIRT1/NF-κB signaling pathway, thereby displaying anti-AD effects.

Multiple investigations have also unveiled the crucial role of SIRT1 in promoting neuronal survival through the deacetylation of p53 (Kim et al. 2007; Hasegawa and Yoshikawa 2008). Initially, p53 was identified as the first non-histone deacetylation target for SIRT1 (Luo et al. 2001). The binding of SIRT1 to p53 at the lysine residue K382 effectively inhibits p53’s nuclear translocation. Consequently, deacetylated p53 relocates to the outer membrane of mitochondria, where it interacts with anti-apoptotic BCL2 protein, leading to the release of the proapoptotic protein BAX. The activation of BAX subsequently triggers the liberation of Cytochrome C from mitochondria to the cytoplasm, instigating a transcription-independent apoptotic cascade orchestrated by p53 (Yi and Luo 2010). Thus, the observed reduction in p53 levels, as well as the diminished acetylation of p53, and the decreased Bax to Bcl-2 ratio following treatment with RSSW, highlight the potential of RSSW in mitigating neuronal apoptosis by modulating the SIRT1/p53 signaling pathway, thereby exhibiting promising anti-AD effects.

The interplay between SIRT1 and AMPK plays a crucial role in regulating energy, metabolism, and aging, as they can mutually enhance each other’s activity (Sung et al. 2015; Chen et al. 2020). It is important to emphasize the significant involvement of the SIRT1/LKB1/AMPK pathway in suppressing senescence (Kauppinen et al. 2013). SIRT1 effectively deacetylates LKB1 at Lys48, thereby activating LKB1 and subsequently promoting AMPK phosphorylation (Lan et al. 2008). The collaboration between Sirt1 and AMPK, both acting as sensors of energy deficit, AMP, and ADP, is particularly intriguing as it facilitates the promotion of autophagy (DiNicolantonio et al. 2022). This process contributes to cellular cleanliness by eliminating damaged proteins and organelles, while also ensuring efficient mitochondrial function and minimal production of oxidants (DiNicolantonio et al. 2022). Consequently, our findings suggested that RSSW had the potential to induce cell autophagy through the SIRT1/AMPK signaling pathway, thereby potentially extending lifespan and ameliorating AD.

Our collaborative research has uncovered the complex molecular mechanisms through which TCM provides neuroprotection against AD. The study robustly demonstrates the effectiveness of the natural herbal formula, RSSW, in mitigating progressive AD by reducing inflammation, retarding aging, inhibiting apoptosis, and modulating the function of Sirt1. To extend the applicability of these findings to a broader patient population, ongoing research is set to include clinical trials on human subjects, particularly those with mild cognitive impairment or in the early stages of AD. These trials will focus on evaluating the safety, tolerability, and preliminary efficacy of RSSW, paving the way for its potential clinical application.

## Conclusions

We have provided compelling evidence for the anti-AD activities of RSSW, which included the attenuation of memory impairments, inhibition of tau hyperphosphorylation, and reduction of β-amyloid plaque deposition in SAMP8 mice. To elucidate the mechanism underlying RSSW’s actions against AD, we employed a comprehensive approach that involved the identification of absorbed components, network pharmacology and molecular docking prediction, and experimental validation. Our findings indicated that RSSW exerts its protective effects through the activation of SIRT1-mediated signaling pathways, specifically the SIRT1/NF-κB, SIRT1/AMPK, and SIRT1/p53 cascades. These pathways have the potential to mitigate cortical tissue inflammation and apoptosis. Consequently, our study highlights the promising therapeutic potential of the traditional Chinese medicine RSSW in the development of novel interventions for AD.

## Consent form

All named authors have agreed to the publication of the work.

## Supplementary Material

Table S1.xlsx

Figure S1 .docx

Table S2.docx

Figure S2.docx

## Data Availability

Data will be made available on request.

## References

[CIT0001] Basheer N, Smolek T, Hassan I, Liu F, Iqbal K, Zilka N, Novak P. 2023. Does modulation of tau hyperphosphorylation represent a reasonable therapeutic strategy for Alzheimer’s disease? From preclinical studies to the clinical trials. Mol Psychiatry. 28(6):2197–2214. doi: 10.1038/s41380-023-02113-z. 37264120 PMC10611587

[CIT0002] Bell L, Whyte A, Duysburgh C, Marzorati M, Van den Abbeele P, Le Cozannet R, Fança-Berthon P, Fromentin E, Williams C. 2022. A randomized, placebo-controlled trial investigating the acute and chronic benefits of American Ginseng (Cereboost^®^) on mood and cognition in healthy young adults, including *in vitro* investigation of gut microbiota changes as a possible mechanism of action. Eur J Nutr. 61(1):413–428. doi: 10.1007/s00394-021-02654-5. 34396468 PMC8783888

[CIT0003] Boaru SG, Borkham-Kamphorst E, Tihaa L, Haas U, Weiskirchen R. 2012. Expression analysis of inflammasomes in experimental models of inflammatory and fibrotic liver disease. J Inflamm. 9(1):49. doi: 10.1186/1476-9255-9-49. PMC3599703 23192004

[CIT0004] Chan YC, Wang MF, Chang HC. 2003. *Polygonum multiflorum* extracts improve cognitive performance in senescence accelerated mice. Am J Chin Med. 31(2):171–179. doi: 10.1142/S0192415X03000862. 12856856

[CIT0005] Chen C, Zhou M, Ge Y, Wang X. 2020. SIRT1 and aging related signaling pathways. Mech Ageing Dev. 187:111215. doi: 10.1016/j.mad.2020.111215. 32084459

[CIT0006] Chen J, Zhou Y, Mueller-Steiner S, Chen LF, Kwon H, Yi S, Mucke L, Gan L. 2005. SIRT1 protects against microglia-dependent amyloid-beta toxicity through inhibiting NF-kappaB signaling. J Biol Chem. 280(48):40364–40374. doi: 10.1074/jbc.M509329200. 16183991

[CIT0007] de Oliveira Zanuso B, de Oliveira Dos Santos AR, Miola VFB, Guissoni Campos LM, Spilla CSG, Barbalho SM. 2022. *Panax ginseng* and aging related disorders: a systematic review. Exp Gerontol. 161:111731. doi: 10.1016/j.exger.2022.111731. 35143871

[CIT0008] Ding M, Ma W, Wang X, Chen S, Zou S, Wei J, Yang Y, Li J, Yang X, Wang H, et al. 2019. A network pharmacology integrated pharmacokinetics strategy for uncovering pharmacological mechanism of compounds absorbed into the blood of Dan-Lou tablet on coronary heart disease. J Ethnopharmacol. 242:112055. doi: 10.1016/j.jep.2019.112055. 31276751

[CIT0009] DiNicolantonio JJ, McCarty MF, Assanga SI, Lujan LL, O’Keefe JH. 2022. Ferulic acid and berberine, via Sirt1 and AMPK, may act as cell cleansing promoters of healthy longevity. Open Heart. 9(1):e001801. doi: 10.1136/openhrt-2021-001801. 35301252 PMC8932268

[CIT0010] Donmez G. 2012. The neurobiology of sirtuins and their role in neurodegeneration. Trends Pharmacol Sci. 33(9):494–501. doi: 10.1016/j.tips.2012.05.007. 22749331

[CIT0011] Gao D, Hao JP, Li BY, Zheng CC, Miao BB, Zhang L, Li YL, Li L, Li XJ, Zhang L. 2023. Tetrahydroxy stilbene glycoside ameliorates neuroinflammation for Alzheimer’s disease via cGAS-STING. Eur J Pharmacol. 953:175809. doi: 10.1016/j.ejphar.2023.175809. 37328043

[CIT0012] Garcia-Just A, Miró L, Pérez-Bosque A, Amat C, Polo J, Pallàs M, Griñán-Ferré C, Moretó M. 2020. Dietary spray-dried porcine plasma prevents cognitive decline in senescent mice and reduces neuroinflammation and oxidative stress. J Nutr. 150(2):303–311. doi: 10.1093/jn/nxz239. 31562503

[CIT0013] Hasegawa K, Yoshikawa K. 2008. Necdin regulates p53 acetylation via Sirtuin1 to modulate DNA damage response in cortical neurons. J Neurosci. 28(35):8772–8784. doi: 10.1523/JNEUROSCI.3052-08.2008. 18753379 PMC6670824

[CIT0014] Hemann MT, Lowe SW. 2006. The p53-Bcl-2 connection. Cell Death Differ. 13(8):1256–1259. doi: 10.1038/sj.cdd.4401962. 16710363 PMC4590992

[CIT0015] Kauppinen A, Suuronen T, Ojala J, Kaarniranta K, Salminen A. 2013. Antagonistic crosstalk between NF-κB and SIRT1 in the regulation of inflammation and metabolic disorders. Cell Signalling. 25(10):1939–1948. doi: 10.1016/j.cellsig.2013.06.007. 23770291

[CIT0016] Kim D, Nguyen MD, Dobbin MM, Fischer A, Sananbenesi F, Rodgers JT, Delalle I, Baur JA, Sui G, Armour SM, et al. 2007. SIRT1 deacetylase protects against neurodegeneration in models for Alzheimer’s disease and amyotrophic lateral sclerosis. EMBO J. 26(13):3169–3179. doi: 10.1038/sj.emboj.7601758. 17581637 PMC1914106

[CIT0017] Kim HJ, Jung SW, Kim SY, Cho IH, Kim HC, Rhim H, Kim M, Nah SY. 2018. Panax ginseng as an adjuvant treatment for Alzheimer’s disease. J Ginseng Res. 42(4):401–411. doi: 10.1016/j.jgr.2017.12.008. 30337800 PMC6190533

[CIT0018] Konecny J, Misiachna A, Hrabinova M, Pulkrabkova L, Benkova M, Prchal L, Kucera T, Kobrlova T, Finger V, Kolcheva M, et al. 2020. Pursuing the complexity of Alzheimer’s disease: discovery of fluoren-9-amines as selective butyrylcholinesterase inhibitors and *N*-methyl-d-aspartate receptor antagonists. Biomolecules. 11(1):3. doi: 10.3390/biom11010003. 33375115 PMC7822176

[CIT0019] Lan F, Cacicedo JM, Ruderman N, Ido Y. 2008. SIRT1 modulation of the acetylation status, cytosolic localization, and activity of LKB1. Possible role in AMP-activated protein kinase activation. J Biol Chem. 283(41):27628–27635. doi: 10.1074/jbc.M805711200. 18687677 PMC2562073

[CIT0020] Lee MS, Yang EJ, Kim JI, Ernst E. 2009. Ginseng for cognitive function in Alzheimer’s disease: a systematic review. J Alzheimers Dis. 18(2):339–344. doi: 10.3233/JAD-2009-1149. 19584437

[CIT0021] Li X, Liu Z, Liao J, Chen Q, Lu X, Fan X. 2023. Network pharmacology approaches for research of Traditional Chinese Medicines. Chin J Nat Med. 21(5):323–332. doi: 10.1016/S1875-5364(23)60429-7. 37245871

[CIT0022] Li Y, Liang W, Guo C, Chen X, Huang Y, Wang H, Song L, Zhang D, Zhan W, Lin Z, et al. 2020. Renshen Shouwu extract enhances neurogenesis and angiogenesis via inhibition of TLR4/NF-κB/NLRP3 signaling pathway following ischemic stroke in rats. J Ethnopharmacol. 253:112616. doi: 10.1016/j.jep.2020.112616. 32007631

[CIT0023] Luo J, Nikolaev AY, Imai S, Chen D, Su F, Shiloh A, Guarente L, Gu W. 2001. Negative control of p53 by Sir2alpha promotes cell survival under stress. Cell. 107(2):137–148. doi: 10.1016/s0092-8674(01)00524-4. 11672522

[CIT0024] Luo Z, Shi J, Jiang Q, Yu G, Li X, Yu Z, Wang J, Shi Y. 2023. Gallic acid enhances anti-lymphoma function of anti-CD19 CAR-T cells *in vitro* and *in vivo*. Mol Biomed. 4(1):8. doi: 10.1186/s43556-023-00122-6. 36871129 PMC9985527

[CIT0025] Luo Z, Yu G, Chen X, Liu Y, Zhou Y, Wang G, Shi Y. 2020. Integrated phytochemical analysis based on UHPLC-LTQ-Orbitrap and network pharmacology approaches to explore the potential mechanism of *Lycium ruthenicum* Murr. for ameliorating Alzheimer’s disease. Food Funct. 11(2):1362–1372. doi: 10.1039/c9fo02840d. 31967149

[CIT0026] Luo Z, Yu G, Han X, Liu Y, Wang G, Li X, Yang H, Sun W. 2021. Exploring the active components of Simotang oral liquid and their potential mechanism of action on gastrointestinal disorders by integrating ultrahigh-pressure liquid chromatography coupled with linear ion trap-orbitrap analysis and network pharmacology. ACS Omega. 6(3):2354–2366. doi: 10.1021/acsomega.0c05680. 33521474 PMC7841926

[CIT0027] Luthra R, Roy A. 2022. Role of medicinal plants against neurodegenerative diseases. Curr Pharm Biotechnol. 23(1):123–139. doi: 10.2174/1389201022666210211123539. 33573549

[CIT0028] Min SW, Cho SH, Zhou Y, Schroeder S, Haroutunian V, Seeley WW, Huang EJ, Shen Y, Masliah E, Mukherjee C, et al. 2010. Acetylation of tau inhibits its degradation and contributes to tauopathy. Neuron. 67(6):953–966. doi: 10.1016/j.neuron.2010.08.044. 20869593 PMC3035103

[CIT0029] Min SW, Sohn PD, Li Y, Devidze N, Johnson JR, Krogan NJ, Masliah E, Mok SA, Gestwicki JE, Gan L. 2018. SIRT1 deacetylates tau and reduces pathogenic tau spread in a mouse model of tauopathy. J Neurosci. 38(15):3680–3688. doi: 10.1523/JNEUROSCI.2369-17.2018. 29540553 PMC5895994

[CIT0030] Parvez MK. 2018. Natural or plant products for the treatment of neurological disorders: current knowledge. Curr Drug Metab. 19(5):424–428. doi: 10.2174/1389200218666170710190249. 28699506

[CIT0031] Ren W, Luo Z, Pan F, Liu J, Sun Q, Luo G, Wang R, Zhao H, Bian B, Xiao X, et al. 2020. Integrated network pharmacology and molecular docking approaches to reveal the synergistic mechanism of multiple components in venenum bufonis for ameliorating heart failure. PeerJ. 8:e10107. doi: 10.7717/peerj.10107. 33194384 PMC7605218

[CIT0032] Shahnur A, Nakano M, Ishihara S, Kakuda N, Miyasaka T, Uchiyama H, Shirai Y, Moniruzzaman M, Saito T, Saido TC, et al. 2021. A potential defense mechanism against amyloid deposition in cerebellum. Biochem Biophys Res Commun. 535:25–32. doi: 10.1016/j.bbrc.2020.12.036. 33340762

[CIT0033] Shannon P, Markiel A, Ozier O, Baliga NS, Wang JT, Ramage D, Amin N, Schwikowski B, Ideker T. 2003. Cytoscape: a software environment for integrated models of biomolecular interaction networks. Genome Res. 13(11):2498–2504. doi: 10.1101/gr.1239303. 14597658 PMC403769

[CIT0034] Singh M, Jindal D, Kumar R, Pancham P, Haider S, Gupta V, Mani S, R R, Tiwari RK, Chanda S. 2023. Molecular docking and network pharmacology interaction analysis of *Gingko biloba* (EGB761) extract with dual target inhibitory mechanism in Alzheimer’s disease. J Alzheimers Dis. 93(2):705–726. doi: 10.3233/JAD-221222. 37066913

[CIT0035] Sung B, Chung JW, Bae HR, Choi JS, Kim CM, Kim ND. 2015. *Humulus japonicus* extract exhibits antioxidative and anti-aging effects via modulation of the AMPK-SIRT1 pathway. Exp Ther Med. 9(5):1819–1826. doi: 10.3892/etm.2015.2302. 26136899 PMC4471809

[CIT0036] Wan L, Cheng Y, Luo Z, Guo H, Zhao W, Gu Q, Yang X, Xu J, Bei W, Guo J. 2015. Neuroprotection, learning and memory improvement of a standardized extract from Renshen Shouwu against neuronal injury and vascular dementia in rats with brain ischemia. J Ethnopharmacol. 165:118–126. doi: 10.1016/j.jep.2015.02.027. 25704930

[CIT0037] Weller J, Budson A. 2018. Current understanding of Alzheimer’s disease diagnosis and treatment. F1000Res. 7:1161. doi: 10.12688/f1000research.14506.1. PMC6073093 30135715

[CIT0038] Wu JS, Zhang FQ, Li ZZ, Jin WY, Shi Y. 2022. Integration strategy of network pharmacology in Traditional Chinese Medicine: a narrative review. J Tradit Chin Med. 42:479–486. 35610020 10.19852/j.cnki.jtcm.20220408.003 PMC9924699

[CIT0039] Yeung F, Hoberg JE, Ramsey CS, Keller MD, Jones DR, Frye RA, Mayo MW. 2004. Modulation of NF-kappaB-dependent transcription and cell survival by the SIRT1 deacetylase. EMBO J. 23(12):2369–2380. doi: 10.1038/sj.emboj.7600244. 15152190 PMC423286

[CIT0040] Yi J, Luo J. 2010. SIRT1 and p53, effect on cancer, senescence and ­beyond. Biochim Biophys Acta. 1804(8):1684–1689. doi: 10.1016/j.bbapap.2010.05.002. 20471503 PMC2989880

[CIT0041] Yu G, Luo Z, Zhou Y, Zhang L, Wu Y, Ding L, Shi Y. 2019. Uncovering the pharmacological mechanism of *Carthamus tinctorius* L. on cardiovascular disease by a systems pharmacology approach. Biomed Pharmacother. 117:109094. doi: 10.1016/j.biopha.2019.109094. 31203131

[CIT0042] Zhang XX, Tian Y, Wang ZT, Ma YH, Tan L, Yu JT. 2021. The epidemiology of Alzheimer’s disease modifiable risk factors and prevention. J Prev Alzheimers Dis. 8(3):313–321. doi: 10.14283/jpad.2021.15.34101789

[CIT0043] Zhao L, Zhang H, Li N, Chen J, Xu H, Wang Y, Liang Q. 2023. Network pharmacology, a promising approach to reveal the pharmacology mechanism of Chinese medicine formula. J Ethnopharmacol. 309:116306. doi: 10.1016/j.jep.2023.116306. 36858276

[CIT0044] Zhou W, Zhang H, Wang X, Kang J, Guo W, Zhou L, Liu H, Wang M, Jia R, Du X, et al. 2022. Network pharmacology to unveil the mechanism of Moluodan in the treatment of chronic atrophic gastritis. Phytomedicine. 95:153837. doi: 10.1016/j.phymed.2021.153837. 34883416

