# Supplementary material for: Integrating network pharmacology and experimental validation to investigate the effects and mechanism of Renshen Shouwu decoction for ameliorating Alzheimer’s disease
Source: Pharm Biol. 2024 Oct 17;62(1):767–80. doi: 10.1080/13880209.2024.2415660 (PMC11488172; doi:10.1080/13880209.2024.2415660)
Supplement: Figure S1 .docx [file IPHB_A_2415660_SM6364.docx]

A

B

C

D

**Fig. S1** Total ion chromatograms of RSSW decoction (A), solvent blank (B), RSSW-treated plasma sample (C) and blank plasma sample (D).
