# Supplementary material for: Integrating network pharmacology and experimental validation to investigate the effects and mechanism of Renshen Shouwu decoction for ameliorating Alzheimer’s disease
Source: Pharm Biol. 2024 Oct 17;62(1):767–80. doi: 10.1080/13880209.2024.2415660 (PMC11488172; doi:10.1080/13880209.2024.2415660)
Supplement: Table S2.docx [file IPHB_A_2415660_SM6363.docx]

Table S2 Molecular docking results

| **Compounds** | Binding energy（kcal/mol） |
| --- | --- |
|  |  |
| ginsenoside Rb2 | -9.6 |
| epicatechin-3-O-gallate | -9.6 |
| kaempferol | -9.4 |
| catechin | -9.2 |
| quercetin | -9.2 |
| 2,3,6-trimethylnaphthalene | -8.5 |
| emodin anthrone | -8.3 |
| ginsenoside Rg4 | -8.2 |
| zingibroside R1 | -8.2 |
