# Supplementary material for: Integrating network pharmacology and experimental validation to investigate the effects and mechanism of Renshen Shouwu decoction for ameliorating Alzheimer’s disease
Source: Pharm Biol. 2024 Oct 17;62(1):767–80. doi: 10.1080/13880209.2024.2415660 (PMC11488172; doi:10.1080/13880209.2024.2415660)
Supplement: Figure S2.docx [file IPHB_A_2415660_SM6362.docx]

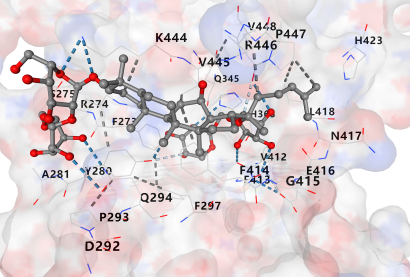

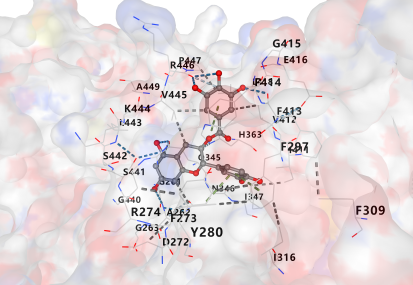

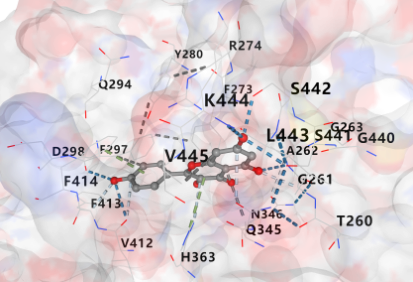


A-Ginsenoside Rb2 B-Epicatechin-3-O-gallate C-Kaempferol


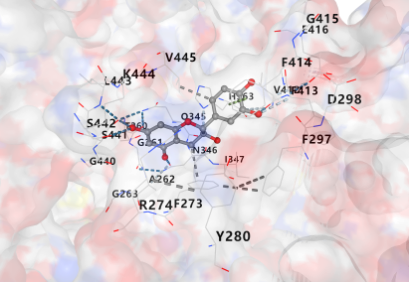

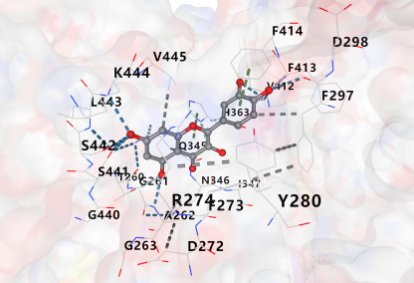

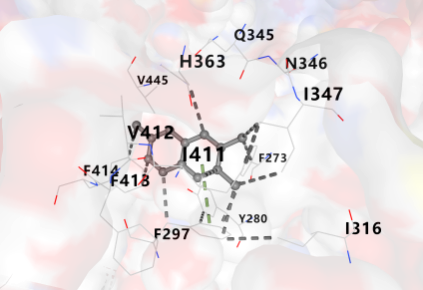


1. Catechin E-Quercetin F- 2,3,6-Trimethylnaphthalene


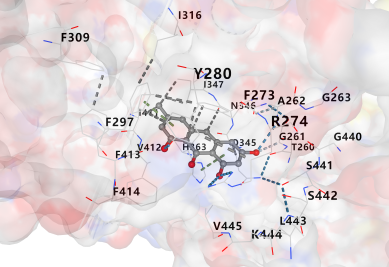

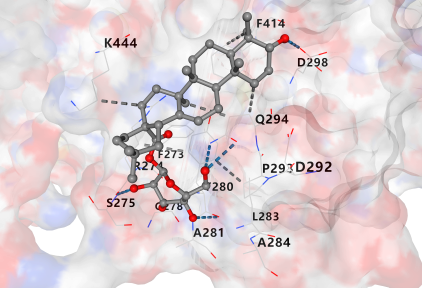

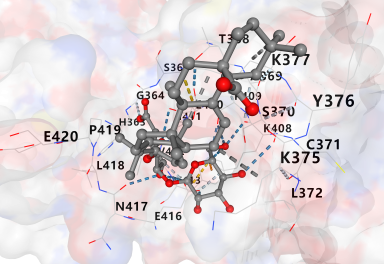


G-Emodin anthrone H-Ginsenoside Rg4 I-Zingibroside R1

**Fig. S2** 3D diagram of molecular docking between components of RSSW and SIRT1
